# The Profiles of Diet- or Exercise-Related Self-Efficacy and Social Support Associated with Insufficient Fruit/Vegetable Intake and Exercise in Women with Abdominal Obesity

**DOI:** 10.3390/nu17152478

**Published:** 2025-07-29

**Authors:** Yanjing Zeng, Qing Long, Yan Jiang, Jieqian Li, Zhenzhen Rao, Jie Zhong, Jia Guo

**Affiliations:** 1 Xiangya School of Nursing, Central South University, 172 Tongzipo Road, Changsha 410013, China; zengyanjing@csu.edu.cn (Y.Z.); longqing226@163.com (Q.L.); 247801001@csu.edu.cn (Y.J.); 227811049@csu.edu.cn (J.L.); 2 Xiangya School of Public Health, Central South University, 172 Tongzipo Road, Changsha 410013, China; zhenzhenrao@csu.edu.cn; 3 School of Nursing, The University of Hong Kong, 5/F, Academic Building, 3 Sassoon Road, Pokfulam, Hong Kong, China

**Keywords:** abdominal obesity, diet, fruit and vegetable intake, exercise, self-efficacy, social support

## Abstract

Background/Objectives: Prioritizing diet- or exercise-related self-efficacy and social support with their interactions may improve the effectiveness of interventions aimed at increasing daily fruit/vegetable intake and exercise, thereby reducing the risk of metabolic disorders in abdominally obese women. This study aimed to identify the profiles of diet- or exercise-related self-efficacy and social support among women with abdominal obesity, examine profiles related to insufficient fruit/vegetable intake and exercise, and explore associating factors of these profiles. Methods: A cross-sectional investigation in central south mainland China collected sociodemographic, anthropometric, and health-related variables, diet-related self-efficacy (Diet-SE) and social support (Diet-SS), exercise-related self-efficacy (Exercise-SE) and social support (Exercise-SS), and daily fruit/vegetable intake and exercise. We used latent profile analysis to identify distinct profiles, and binary logistic regression to examine the profiles’ behaviors and associating factors. Results: A total of 327 abdominally obese women were categorized into four profiles of Diet-SE and Diet-SS, and five profiles of Exercise-SE and Exercise-SS. Women in the Diet Dual-Low Group were associated with insufficient daily fruits/vegetables intake. Women in the Exercise Dual-Low Group or Exercise-SS Medium–Low Group were more likely to engage in insufficient daily exercise. Conclusions: Our findings align with previous evidence that women with low diet- or exercise-related self-efficacy and social support are at increased risk for insufficient daily fruit/vegetable intake or exercise. Additionally, medium Exercise-SS is associated with insufficient exercise behaviors, suggesting that interventions targeting healthy exercise should be initiated earlier among women with medium Exercise-SS, rather than waiting for it to decline to low level.

## 1. Introduction

The global prevalence of obesity was 14% in 2020 and is projected to be 24% by 2035, imposing heavy healthcare costs [1]. In most countries and regions, the prevalence of obesity is higher among adult women compared to men [2]. Compared to peripheral obesity, abdominal obesity is more closely linked to metabolic disorders [3]. The unhealthy lifestyle associated with abdominal obesity is characterized by low intakes of fresh fruits and vegetables, as well as low levels of physical activity [4,5]. The World Health Organization guidelines recommend that adults consume at least 400 g (equivalent to five servings) of fruits and vegetables and engage in at least 30 min of moderate-intensity aerobic physical activity daily to reduce the risk of obesity [6,7]. However, over half of the general adult population in China demonstrates inadequate intake of fruits and vegetables [8], while approximately one-quarter of the global adult population does not engage in sufficient daily physical activity [9].

According to the Social Cognitive Theory (SCT), self-efficacy and social support are crucial mediators in behavior change processes that necessitates sustained effort [10]. Specifically, compared to general self-efficacy and social support, diet- or exercise-related self-efficacy and social support are more relevant to dietary or exercise behavior change, which need to be targeted in intervention development [11,12]. Numerous SCT-based lifestyle interventions have been designed to modify the aforementioned unhealthy lifestyles among obese adults [13]. However, the most recent umbrella review shows that only 59% of lifestyle interventions effectively promote fruit and vegetable intake [14]. Another meta-analysis indicates a small overall effect size of 0.04 for promoting physical activity [15]. While both self-efficacy and social support have shown small effects in behavior change, it might be possible that their combination could potentially strengthen the efficacy of SCT-based interventions [16,17].

Currently, there are interactions between self-efficacy and social support. Bandura’s theoretical framework posits that higher levels of social support may enhance self-efficacy by mitigating emotional arousal, gaining vicarious experiences, and receiving verbal backup [18]. Research that used the SCT as a theoretical framework to explore the influencing factors of dietary habits suggests a potential relationship between different levels of self-efficacy and the utilization of social support on behaviors [19]. Moreover, a study in young adults found that social support not only directly influences exercise behaviors but also operates through self-efficacy [20]. Current interventions aimed at improving fruit and vegetable intake or exercise often overlook the interactive influence of these two mediators, which may explain the previously noted small effect sizes of these interventions. It is essential to consider the potential interactions between diet- or exercise-related self-efficacy and social support before the development of tailored interventions for women with abdominal obesity.

Conventional variable-level analysis evaluates individual self-efficacy and social support in isolation, which can only delineate their respective contributions to predictive relationships with fruit and vegetable intake or exercise [21,22]. As a person-centered algorithm, latent profile analysis (LPA) classifies individuals into unobserved groups based on similar patterns [23]. LPA can provide nuanced phenotypes of self-efficacy and social support for individuals, while variable-level analysis cannot. For instance, some individuals may experience insufficient fruit and vegetable intake or exercise due to low self-efficacy, while others may possess high self-efficacy yet lack adequate social support, resulting in insufficient external encouragement. Therefore, this study aims to (a) identify the profiles of diet- or exercise-related self-efficacy and social support among women with abdominal obesity; (b) examine which profiles were related to insufficient fruit/vegetable intake and exercise respectively; (c) explore the factors associated with the identified profiles concerning insufficient fruit/vegetable intake or exercise.

## 2. Materials and Methods

### 2.1. Study Design

This cross-sectional observational study was reported following the Strengthening the Reporting of Observational Studies in Epidemiology (STROBE) Statement.

### 2.2. Setting

The study was conducted between 1 July 2021 and 30 June 2023 in Changsha, the capital city of Hunan province, located in the central south of mainland China with a GDP of USD 198.1 billion in 2023. The research sites included two tertiary hospitals located in the eastern and western regions of the city, both designed to serve the entire population of the city. Participants were recruited from the Adults Health Management Centers at the research sites and the Children’s Health Management Centers, where mothers bring their children for physical examinations.

### 2.3. Participants

The inclusion criteria were as follows: (1) women defined by biological sex; (2) aged 18 years and above (including 18 years); (3) with a waist circumference of 80 cm or more; (4) with the youngest child aged between 1 and 12 years; (5) being able to read and speak Mandarin; (6) joining the study voluntarily. Exclusion criteria were: (1) current pregnancy; (2) having an acute or life-threatening illness (e.g., renal failure) which may affect the accuracy of measurements.

The sample size was calculated using PASS 15.0 software. A power analysis was conducted using logistic regression with an alpha of 0.05, a power (1−β) of 0.80, P0(Y = 1|X = 0) of 0.471, an odds ratio of 5.03, an R^2^ of 0.08, and a percentage of N(X1 = 1) of 6. The minimum required sample size was determined to be 278 participants. Considering an estimated non-response rate of 15%, based on our previous investigation within this population, we decided to recruit 327 participants.

### 2.4. Data Collection

Women contacted the research team via the phone numbers of the research assistants provided on the flyers and posters displayed in the research sites. Once the women expressed interest in participating, trained research assistants who worked as nurses at research sites verified their eligibility, including self-measurement of waist circumference, following standard guidelines. Eligible participants were informed of their right to withdraw from the study. After the women signed the informed consent, they were required to complete a set of paper-based questionnaires in a private room at the research site. Research assistants could explain questions regarding the questionnaire and check each completed questionnaire for missing items. Participants were compensated with 10 RMB (1.55 dollars) for their time. The research was approved by the Ethics Committee of Xiangya School of Nursing, Central South University (2019002).

### 2.5. Measurement

The set of questionnaires collected data on sociodemographic and anthropometric information, health-related variables, diet- and exercise-related self-efficacy and social support status, and daily fruit/vegetable intake and exercise.

A self-designed sociodemographic sheet was used to collect data on age, residence, ethnicity, marital status, education, occupation, monthly income, and age of the youngest child. Anthropometric data included body mass index (BMI) and waist circumference. BMI was calculated by dividing body weight (in kilograms) by height squared (in meters). According to the WHO approach [24], waist circumference was measured at the midpoint between the highest point of the iliac crest and the lowest rib. Health-related variables included the number of pregnancies, any chronic diseases (e.g., hyperglycemia, cardiovascular disease, and hypertension, etc.), and family history of chronic diseases (including diabetes, cardiovascular disease, and hypertension).

The Health-Related Diet and Exercise Self-Efficacy Scale was used to assess participants’ diet- and exercise-related self-efficacy [11]. Each subscale, for diet and exercise, contains four items rated on a 5-point scale: 0 (I’m sure I cannot), 1 (mostly I cannot), 2 (I don’t know), 3 (mostly I can), or 4 (I’m sure I can). Total scores for each subscale range from 0 to 16, with higher scores indicating higher levels of diet- or exercise-related self-efficacy. We categorize the two subscales into three levels: 0–5 as low, 6–11 as medium, and 12–16 as high, which are used only to name and interpret the identified profiles.

The Health-Related Diet and Exercise Social Support Scale was used to assess participants’ diet- and exercise-related social support [12]. Each subscale, for diet and exercise, contains six items. The diet subscale is scored on a 4-point scale from 1 (Never) to 4 (Always), with total scores between 6 and 24; higher scores indicate higher levels of diet-related social support. The exercise subscale is scored on a 5-point scale from 0 (Never) to 4 (Very Often), with total scores between 0 to 24; higher scores indicate higher levels of exercise-related social support. We categorize the two subscales into low (6–12 for diet; 0–8 for exercise), medium (13–18 for diet; 9–16 for exercise), and high (19–24 for diet; 17–24 for exercise) levels, which are used only to name and interpret the identified profiles as well.

The Health-Related Diet and Exercise Self-Efficacy Scale and the Health-Related Diet and Exercise Social Support Scale were originally developed to assess self-efficacy and perceived social support specific to health-related eating and exercise behaviors in American populations. Subsequently, the original scales were simplified, reducing the number of items from 61 to 4 for diet-related self-efficacy, from 12 to 4 for exercise-related self-efficacy, from 23 to 6 for diet-related social support, and from 20 to 6 for exercise-related social support. The simplified versions were translated, culturally adapted, and validated in China, demonstrating good internal consistency with Cronbach’s alpha coefficients of 0.86 for diet-related self-efficacy, 0.91 for exercise-related self-efficacy, 0.86–0.89 for diet-related social support, and 0.87–0.92 for exercise-related social support [25,26]. In the present study, the Cronbach’s alpha coefficients for the Chinese version of the Diet and Exercise Self-Efficacy Scale were 0.872 and 0.892, respectively, while those for the Chinese version of the Diet and Exercise Social Support Scale were 0.592 and 0.848, respectively.

Daily fruit/vegetable intake and exercise were assessed using an adapted item from the Chinese Diabetes Risk Questionnaire. This questionnaire has been validated in Chinese adults and exhibits a retest reliability of 0.988, a positive predictive value of 57%, a negative predictive value of 78%, and a sensitivity of 73% [27]. The questions are “Do you eat five servings of vegetables or fruits every day? (No or Yes)” and “Do you usually do some physical activity such as brisk walking for at least 30 min each day? (No or Yes)”. Answering ‘No’ indicates insufficient daily fruit/vegetable intake or exercise. In the present study, the intraclass correlation coefficients (ICCs), indicating the 1-week test–retest reliability of the fruit and vegetable intake and physical activity items among 23 participants, were 0.889 and 0.796, respectively.

### 2.6. Statistical Analysis

Latent profile analysis was conducted using Mplus 7.4 (Muthén & Muthén, Los Angeles, CA, USA). Continuous scores of diet- or exercise-related self-efficacy and social support were included as indicators. Four fit indices, the Akaike Information Criterion (AIC), the Bayesian Information Criterion (BIC), the value of Sample Size-Adjusted BIC (aBIC), and the entropy test were used to determine the optimal number of profiles. Lower values of AIC, BIC, and aBIC indicate better model fit, while higher entropy (close to 1) represents better separation between profiles [28]. The Lo–Mendell–Rubin likelihood ratio test (LMR) and the bootstrapped likelihood ratio test (BLRT) were used for model comparison. Significant *p*-values on LMR and BLRT support the k number of profiles rather than the k-1 profiles [28]. We tested between 1 and 5 profiles to identify the best-fitting model based on fit indices and the interpretability of profiles.

IBM SPSS 24.0 (IBM Corp., Armonk, NY, USA) was used for the rest of the data analyses. Continuous variables were presented as medians with interquartile ranges or means with standard deviations, while categorical variables were reported as frequency counts with percentages. Missing data were replaced by regression imputation. The number of missing data for most variables ranged from 0 to 3, which is negligible, while the number of missing data for “the number of pregnancies” was 19. We used binary logistic regression to examine which profiles of diet- or exercise-related self-efficacy and social support are related to insufficient fruit/vegetable intake and exercise. Sociodemographic, anthropometric, and health-related variables were adjusted in the models. We used backward-LR selection in the multivariable logistic regression to identify associated factors of the profile concerning insufficient daily fruit/vegetable intake and exercise. *p*-values < 0.05 were considered statistically significant.

## 3. Results

### 3.1. Sociodemographic and Clinical Characteristics

A total of 331 participants completed the questionnaire. After excluding 4 unreliable responses due to more than 20% missing items, 327 questionnaires were included in the analyses, resulting in a response rate of 98.8%. The median age (IQR) of the 327 women with abdominal obesity was 36.00 (33.00, 40.00) years. Approximately 73.4% resided in urban areas and 75.2% reported a monthly income exceeding 233 dollars (~233 dollars, which is considered lower-middle income in China). The median waist circumference was 86.00 (82.00, 93.00) cm, and only 42.8% of participants had a BMI lower than 24.0 kg/m^2^. Over half of the participants (50.5%) reported having no chronic diseases. The characteristics of the sample are presented in Table 1.

### 3.2. Daily Fruit/Vegetable Intake and Exercise Behaviors

More than half of the women did not meet the recommended daily intake of five servings of fruits and vegetables (51.7%) or engage in 30 min of daily exercise (52.0%), both of which are considered unhealthy lifestyle behaviors.

### 3.3. Latent Profile Analysis of Diet-Related Self-Efficacy (Diet-SE) and Social Support (Diet-SS)

**Figure 1:**
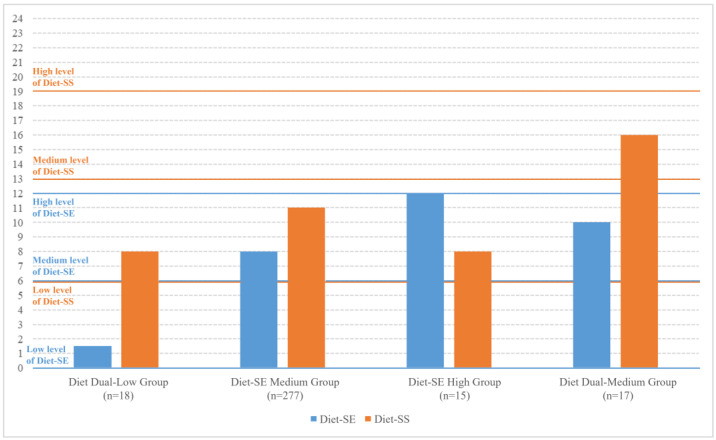
The distribution of four potential profiles displays the median of diet-related self-efficacy and social support.

The median scores for diet-related self-efficacy and social support were 8.00 (IQR = 5.00–10.00) and 11.00 (IQR = 10.00–12.00), respectively. The four-profile model was preferred, as it exhibited the lowest AIC and aBIC values, an entropy greater than 0.8, and significant LMR and BLRT results (Table 2). Figure 1 illustrates the distribution of the four potential profiles. The largest profile (n = 277, 84.7%) exhibited medium Diet-SE and low Diet-SS scores, while the smallest profile (n = 15, 4.6%) displayed high Diet-SE but low Diet-SS scores. The second smallest profile (n = 17, 5.2%) had both medium Diet-SE and Diet-SS scores, and the third smallest profile (n = 18, 5.5%) showed both low Diet-SE and Diet-SS scores (Shown in Supplementary Table S1).

### 3.4. Diet Dual-Low Group Associated with Less than Five Servings of Daily Fruit/Vegetable Intake

The Diet Dual-Low Group (OR = 0.129, 95% CI = 0.025–0.672, adjusted) consumed significantly less than five servings of daily fruits and vegetables compared to the Diet Dual-Medium Group. (Shown in Table 3). Furthermore, the Diet Dual-Low Group also consumed significantly less than five servings of daily fruits and vegetables when compared to the Diet-SE Medium Group (OR = 0.174, 95% CI = 0.045–0.673, adjusted). (Shown in Supplementary Table S2). In summary, the Diet Dual-Low Group demonstrated a higher likelihood of consuming less than five servings of daily fruits and vegetables.

### 3.5. Associating Factors of the Diet Dual-Low Group

As shown in Table 4, participants who are married (OR = 0.061, 95% CI = 0.007–0.525) were less likely to belong to the Diet Dual-Low Group. Participants with a monthly income of less than 233 dollars (OR = 4.735, 95% CI = 1.702–13.171) had higher odds of being classified in the Diet Dual-Low Group.

### 3.6. Latent Profile Analysis of Exercise-Related Self-Efficacy (Exercise-SE) and Social Support (Exercise-SS)

**Figure 2:**
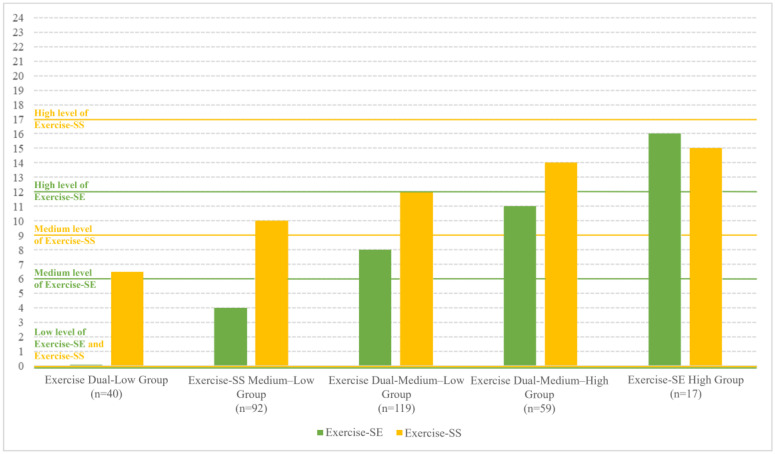
The distribution of five potential profiles displays the median of exercise-related self-efficacy and social support.

The median scores for exercise-related self-efficacy and social support were 7.00 (IQR = 4.00–9.00) and 12.00 (IQR = 8.00–14.00), respectively. The five-profile model was determined to be the most suitable, characterized by the lowest AIC, BIC, and aBIC values, the highest entropy, and significant LMR and BLRT results (Table 2). Figure 2 illustrate the distribution of the five potential profiles. The largest profile (n = 119, 36.4%) had both medium–low Exercise-SE and Exercise-SS scores, while the second largest profile (n = 92, 28.1%) had low Exercise-SE and medium–low Exercise-SS scores. The smallest profile (n = 17, 5.2%) exhibited high Exercise-SE and medium–high Exercise-SS scores, the second smallest profile (n = 40, 12.2%) displayed both low Exercise-SE and Exercise-SS scores, and the third smallest profile (n = 59, 18.0%) had both medium–high Exercise-SE and Exercise-SS scores (Shown in Supplementary Table S1).

### 3.7. Exercise Dual-Low Group and Exercise-SS Medium–Low Group Associated with Less than 30 Min of Daily Exercise

The Exercise Dual–Low Group (OR = 0.046, 95% CI = 0.010–0.213, adjusted) and the Exercise-SS Medium–Low Group (OR = 0.136, 95% CI = 0.037–0.495, adjusted) were significantly associated with engaging in less than 30 min of daily exercise compared to the Exercise-SE High Group. (Shown in Table 5). Furthermore, the Exercise Dual-Low Group (OR = 0.137, 95% CI = 0.047–0.397, adjusted) and the Exercise-SS Medium–Low Group (OR = 0.397, 95% CI = 0.214–0.736, adjusted) were significantly associated with exercising less than 30 min daily when compared to the Exercise Dual-Medium–Low Group (shown in Supplementary Table S3). In summary, both the Exercise Dual-Low Group and the Exercise-SS Medium–Low Group were less likely to achieve 30 min of daily exercise.

### 3.8. Associating Factors of the Exercise Dual-Low Group and the Exercise-SS Medium–Low Group

According to Table 4, participants without any chronic disease (OR = 0.506, 95% CI = 0.316–0.809) were less likely to belong to the Exercise Dual-Low Group and the Exercise-SS Medium–Low Group. Conversely, participants with part-time job or no job (OR = 0.602, 95% CI = 0.364–0.994) or with a monthly income of less than 233 dollars (OR = 0.541, 95% CI = 0.304–0.964) had lower odds of being classified in the Exercise Dual-Low Group and the Exercise-SS Medium–Low Group.

## 4. Discussion

This study identified four distinct profiles of Diet-SE and Diet-SS, and five distinct profiles of Exercise-SE and Exercise-SS, among women with abdominal obesity. The Diet Dual-Low Group was less likely to meet the recommended daily intake of five servings of fruits and vegetables. The Exercise Dual-Low Group and the Exercise-SS Medium–Low Group reported a lower likelihood of engaging in 30 min of daily exercise. Our primary findings align with previous evidence that women with low diet- or exercise-related self-efficacy and social support are at increased risk for insufficient daily fruit and vegetable intake or exercise. Our findings extend this understanding by revealing medium Exercise-SS may be associated with insufficient exercise behaviors.

There is substantial potential to enhance daily fruit and vegetable intake and exercise among Chinese women with abdominal obesity. Over half of the participants failed to meet the recommended daily intake of five servings of fruits and vegetables (51.7%) or engage in 30 min of daily exercise (52.0%). Similarly, 41.5% of Chinese working women had insufficient fruit and vegetable consumption in 2018 [29]. What’s worse is that a U.S. survey shows only 14.5% of adult women meet recommendations for fruit intake and 12.4% for vegetable intake [30]. These results not only confirm the association between abdominal obesity-related unhealthy lifestyles and insufficient fruit and vegetable intake [4] but also highlight the unhealthy dietary patterns prevalent in developed countries, such as high consumption of processed foods and sugar-sweetened beverages, coupled with low intake of whole grains, fruits, and vegetables [31]. Although fruits and vegetables are generally more affordable than processed foods and meat in China, limited nutrition knowledge, individual dietary preferences, and the rising cost of living may still restrict their consumption among low socioeconomic status populations living in rural areas [29]. In terms of physical activity, the global prevalence of physical inactivity among women decreases with national income levels: 41.6% in high-income, 30.1% in middle-income, and 18.8% in low-income countries [32]. The difference between our research and global surveys reflects both urbanization effects in China [33] and abdominal obesity-related sedentary behaviors [5].

Our study identified four distinct profiles in Diet-SE and Diet-SS: the Diet Dual-Low Group (5.5%) had the lowest intake of fruits and vegetables, while the largest proportion was the Diet-SE Medium Group (84.7%). Additionally, there were five profiles in Exercise-SE and Exercise-SS: the Exercise Dual-Low Group (12.2%) and the Exercise-SS Medium–Low Group (28.1%) both had the least amount of daily exercise. Our findings highlight the heterogeneity in Diet-SE, Diet-SS, Exercise-SE, and Exercise-SS among Chinese women with abdominal obesity, confirming the mutual interaction between self-efficacy and social support in SCT [18], while also providing novel insights, such as the identification of a Diet SE High Group. The proportion of Americans with low Diet-SE (58.9%), low Exercise-SE, and low-to-medium Exercise-SS (9% to 44.9%) was higher than what was observed in our research [34,35], which can be explained by behavioral differences caused by urbanization [30,33].

The implications of these profiles become particularly evident when examining their relationship with health behaviors. Our findings are aligned with prior evidence suggesting that higher levels of Diet-SE and Diet-SS are correlated with greater fruit and vegetable intake [36,37], and higher levels of Exercise-SE and Exercise-SS are associated with increased physical activity [38]. The theoretical foundations showed that internal self-efficacy towards diet and exercise indicates individuals’ beliefs in their ability to make healthier dietary and exercise choices [36,38], while external social support promotes encouragement and influences healthier eating and exercising habits [37,38]. Unlike fruit and vegetable consumption, medium–low Exercise-SS was associated with insufficient exercise. Given that adherence rates for exercise tend to be lower than those for a healthy diet, we speculate that exercise requires overcoming greater intrinsic behavioral inertia [39]. Medium levels of Exercise-SS may not be enough to motivate individuals, suggesting the need for either medium levels of Exercise-SE in collaboration or higher levels of Exercise-SS [40].

Our study identified marital status and monthly income as significant factors associated with distinct profiles of Diet-SE and Diet-SS in Chinese women. First, compared to single women, married women were less likely to belong to the Diet Dual-Low Group. It aligns with the social protection hypothesis, which posits that marriage constitutes a close relationship that can enhance social support and expand social networks [41]. As a mediator in the interaction between social support and dietary behavior, self-efficacy is positively influenced by increased social support [19]. Additionally, women with a monthly income of less than 233 dollars had higher odds of being classified in the Diet Dual-Low Group. Higher income may act as an incentive, promoting positive attitudes and life satisfaction in their environment [42]. Conversely, lower income can result in a less supportive social network, leading to fewer resources and increased stress [43].

In this study, women without chronic disease were less likely to belong to the Exercise Dual-Low Group and the Exercise-SS Medium–Low Group. This may be because women in better health tend to exhibit greater psychological confidence in adopting healthy lifestyle behaviors and are more likely to receive support from their social surroundings [44]. Interestingly, women with part-time or no job (compared to full-time jobs), and those with a monthly income of less than 233 dollars (compared to more than 233 dollars), had lower odds of being classified in the Exercise Dual–Low Group and the Exercise-SS Medium–Low Group. Generally, full-time jobs often offer more stable financial and social resources, leading to more positive attitudes and supportive environments for exercise [45]. Although women with higher incomes generally report greater self-efficacy and social support [46], increased work demands may reduce their available leisure time and energy, limiting opportunities to build internal confidence and perceive social support for physical activity [47]. In contrast, part-time employed or unemployed women in China are more likely to engage in community-based leisure-time physical activities (e.g., walking, jogging, square dancing), which provide opportunities for exercise and strong social networks that enhance self-efficacy and social support towards exercise [48]. This pattern is further supported by the widespread availability of urban green spaces, offering free and inclusive access to physical activity, particularly benefiting low-income groups [49].

Moreover, this discrepancy about the monthly income association between exercise and diet may arise from the higher costs of fruits and vegetables compared to exercise [50]. In addition to the aforementioned socio-environmental factors, low-income populations may rely more on active transportation such as walking and cycling [51] and are more likely to perform manual labor [52], both of which may enhance their confidence and perceived support for physical activity.

### 4.1. Implication for Research and Practice

This study has several implications for future research and clinical practice. Theoretically, our findings provide concrete evidence regarding the significance of social cognitive constructs: higher profiles of diet- or exercise-related self-efficacy and social support are associated with better health behaviors. Future research could engage more social cognitive constructs (e.g., intentions, barriers, and social norms) when exploring useful strategies to promote healthy lifestyle behaviors. Furthermore, future research could investigate other influencing factors related to lifestyle behaviors among women with medium-to-high levels of diet- or exercise-related self-efficacy and social support. To accurately capture the nuances and actual levels of dietary and exercise behaviors, future studies are encouraged to employ more robust and validated scales or objective measures (e.g., Food Frequency Questionnaire and accelerometers).

Clinically, healthcare providers can early identify women with abdominal obesity who are at high risk of insufficient daily fruit/vegetable intake or exercise. Considering that the directly used behavioral measurement tools, the Food Frequency Questionnaire and the International Physical Activity Questionnaire, are both recall-based and inefficient [53,54], we therefore recommend the routine screening of individuals with low Diet-SE and Diet-SS, or low Exercise-SE and low-to-medium Exercise-SS in community settings or at medical examination centers, as these scales with a streamlined number of items are more practical. This should be conducted alongside an evaluation of employment status, monthly income, and chronic disease status. This approach could facilitate the earlier initiation of interventions targeting unhealthy diet or exercise behaviors through assessing low diet- or exercise-related self-efficacy and social support, and especially medium Exercise-SS. Such targeted measures could alleviate the burden and inconvenience for women with above-average levels of diet- or exercise-related self-efficacy and social support while enhancing cost-effectiveness in healthcare economics.

### 4.2. Limitation

There are several limitations to be noted. First, the participants were recruited through convenience sampling in one city, which is less generalizable to women living in rural areas. Moreover, the participants were only mothers of children aged 1–12 years, which narrows the applicability of the findings to a specific group of women with abdominal obesity. Therefore, future studies with multicenter designs, larger and more diverse samples are required to enhance the generalizability of the findings. Second, we collected data during the COVID-19 pandemic, during which lockdown policies were enforced. Due to the decreased frequency of purchasing fresh food and exercising outside and the higher possibility of social isolation, women might have experienced less confidence to engage in healthy lifestyles, and were maybe less likely to gain support from others. Consequently, the high percentage of insufficient fruit/vegetable intake and exercise observed in this study may not fully represent the usual patterns. Therefore, the results should be interpreted cautiously in terms of women’s behaviors internationally.

Third, the Cronbach’s alpha of the Chinese version of the Diet Social Support Scale used in this study was 0.592, which is very close to 0.6, the acceptable range for internal consistency reliability. The reduced reliability in the current study may be attributed to the restricted sample, which only included women with abdominal obesity, whereas the previously validated Chinese version of the Diet Social Support Scale was tested in more diverse populations encompassing both adult men and women without restrictions regarding obesity status. A population-specific instrument may be more appropriate to assess diet-related social support for the participants in this study. Fourth, given that previous research has shown that maintaining an adequate daily intake of fruits and vegetables and engaging in regular physical activity can contribute to weight loss among obese individuals, we employed single items with acceptable reliability and validity to assess these two specific behaviors. However, this measurement approach may not fully capture the complexity and variability of these behaviors. Lastly, while the correlational study design provided some implications into causality, the causal relationships among the variables should be explored further.

## 5. Conclusions

Among Chinese women with abdominal obesity, low Diet-SE and Diet-SS are correlated with insufficient fruit and vegetable intake, while low Exercise-SE and low-to-medium Exercise-SS are associated with insufficient daily exercise. These findings facilitate the early identification of women at high risk of insufficient daily fruit and vegetable intake and exercise. These findings revealed a critical nuance in healthy behavior interventions: while women with low Diet-SE, Diet-SS, and Exercise-SE require targeted interventions to address foundational barriers, those with medium Exercise-SS also demonstrate a need for early engagement to prevent declines in physical activity.

## Figures and Tables

**Table 1:** The characteristics of participants. (n = 327).

Variables	n (%)	M (IQR ^a^)
Age, years		36.00 (33.00, 40.00)
Residence		
Urban	240 (73.4)	
Rural	87 (26.6)	
Ethnicity		
Han Chinese	300 (91.7)	
Minority	27 (8.3)	
Marital status		
Married	322 (98.5)	
Single	5 (1.5)	
Education		
9 years or less	86 (26.3)	
10 years or more	241 (73.7)	
Occupation		
Part-time job or no job	219 (67.0)	
Full-time job	108 (33.0)	
Monthly income		
≤1500 RMB (233 dollars)	81 (24.8)	
>1500 RMB (233 dollars)	246 (75.2)	
Number of pregnancies		2.00 (2.00, 3.00)
Age of the youngest child		
1–5 years	123 (37.6)	
6–12 years	204 (62.4)	
WC ^b^, cm		86.00 (82.00, 93.00)
BMI ^c^		
<24.0	140 (42.8)	
24.0–27.9	125 (38.2)	
≥28.0	62 (19.0)	
With any chronic disease		
No	165 (50.5)	
Yes	162 (49.5)	
Family history of diabetes		
No	267 (81.7)	
Yes	60 (18.3)	
Family history of CVD ^d^		
No	275 (84.1)	
Yes	52 (15.9)	
Family history of hypertension		
No	192 (58.7)	
Yes	135 (41.3)	

Note: Missing data for age (n = 2), residence (n = 3), marital status (n = 1), education (n = 1), occupation (n = 2), monthly income (n = 1), number of pregnancies (n = 19), and with any chronic disease (n = 2). ^a^ IQR, interquartile ranges. ^b^ WC, waist circumference. ^c^ BMI, body mass index. ^d^ CVD, cardiovascular disease.

**Table 2:** The fit indices for 1–5 profiles of two models.

Variables	Profiles	AIC ^a^	BIC ^b^	aBIC ^c^	LMR ^d^	BLRT ^e^	Entropy	Category Probability	Case Number
Diet-related self-efficacy and social support	1	3255.622	3270.782	3258.094	— —	— —	— —	1	327
	2	3238.975	3265.505	3243.301	*p* < 0.01	*p* < 0.01	0.916	0.963/0.037	315/12
	3	3234.859	3272.759	3241.039	*p* = 0.257	*p* = 0.05	0.780	0.107/0.841/0.052	35/275/17
	4	3229.831	3279.101	3237.865	*p* < 0.05	*p* < 0.05	0.821	0.046/0.055/0.847/0.052	15/18/277/17
	5	3230.968	3291.607	3240.856	*p* = 0.612	*p* = 0.375	0.722	0.768/0.055/0.07/0.067/0.040	251/18/23/22/13
Exercise-related self-efficacy and social support	1	3740.222	3755.382	3742.694	— —	— —	— —	1	327
	2	3666.209	3692.738	3670.535	*p* < 0.01	*p* < 0.01	0.561	0.529/0.471	173/154
	3	3649.924	3687.824	3656.104	*p* = 0.251	*p* < 0.01	0.671	0.572/0.080/0.349	187/26/114
	4	3645.926	3695.195	3653.960	*p* = 0.729	*p* = 0.146	0.779	0.125/0.119/0.443/0.312	41/39/145/102
	5	3579.451	3640.090	3589.339	*p* < 0.01	*p* < 0.01	0.918	0.122/0.281/0.364/0.180/0.052	40/92/119/59/17

Note: ^a^ AIC: the Akaike information criterion. ^b^ BIC: the Bayesian information criterion. ^c^ aBIC: the value of Sample Size-Adjusted BIC. ^d^ LMR: the Lo–Mendell–Rubin likelihood ratio test. ^e^ BLRT: the bootstrapped likelihood ratio test.

**Table 3:** Binary logistic regression on insufficient daily fruit/vegetable intake.

Variables	*p*	OR	95% CI
Profiles of diet-SE and diet-SS			
Diet Dual-Low Group	0.015	0.129	(0.025, 0.672)
Diet-SE Medium Group	0.490	0.693	(0.244, 1.964)
Diet-SE High Group	0.712	1.340	(0.284, 6.319)
(Ref. Diet Dual-Medium Group)			
Age	0.248	1.028	(0.981, 1.078)
Residence			
Urban	0.527	1.213	(0.667, 2.206)
(Ref. Rural)			
Ethnicity			
Han Chinese	0.823	1.104	(0.463, 2.632)
(Ref. Minority)			
Marital status			
Married	0.864	0.841	(0.115, 6.145)
(Ref. Single)			
Education			
9 years or less	0.082	0.546	(0.276, 1.080)
(Ref. 10 years or more)			
Occupation			
Part-time job or no job	0.262	1.383	(0.785, 2.438)
(Ref. Full-time job)			
Monthly income			
≤233 dollars	0.256	1.441	(0.767, 2.706)
(Ref. > 233 dollars)			
Number of pregnancies	0.187	0.863	(0.693, 1.074)
Age of the youngest child			
1–5 years	0.484	0.825	(0.482, 1.413)
(Ref. 6–12 years)			
WC ^a^	0.921	1.002	(0.968, 1.036)
BMI ^b^			
<24.0	0.972	0.987	(0.461, 2.112)
24.0–27.9	0.131	0.573	(0.278, 1.179)
(Ref. ≥ 28.0)			
With any chronic disease			
No	0.013	1.852	(1.140, 3.008)
(Ref. Yes)			
Family history of diabetes			
No	0.301	0.723	(0.392, 1.336)
(Ref. Yes)			
Family history of CVD ^c^			
No	0.836	0.932	(0.481, 1.808)
(Ref. Yes)			
Family history of hypertension			
No	0.509	1.182	(0.719, 1.943)
(Ref. Yes)			

Note: The model adjusted for sociodemographic, anthropometric, and health-related variables. ^a^ WC, waist circumference. ^b^ BMI, body mass index. ^c^ CVD, cardiovascular disease.

**Table 4:** The stepwise multivariable logistic regressions on diet- or exercise-related self-efficacy and social support profiles.

Outcome *	Variables	*p*	OR	95% CI
Profiles of Diet-SE and Diet-SS	Ethnicity			
	Han Chinese	0.058	0.278	(0.074, 1.042)
	(Ref. Minority)			
	Marital status			
	Married	0.011	0.061	(0.007, 0.525)
	(Ref. Divorced/widowed)			
	Monthly income			
	≤233 dollars	0.003	4.735	(1.702, 13.171)
	(Ref. > 233 dollars)			
Profiles of Exercise-SE and Exercise-SS	Occupation			
	Part-time job or no job	0.047	0.602	(0.364, 0.994)
	(Ref. Full-time job)			
	Monthly income			
	≤233 dollars	0.037	0.541	(0.304, 0.964)
	(Ref. > 233 dollars)			
	With any chronic disease			
	No	0.004	0.506	(0.316, 0.809)
	(Ref. Yes)			

Note: * We recoded these profiles into binary outcome variables for multivariable logistic regression analyses. For the diet-related profiles, the Diet Dual-Low Group was categorized as the ‘poorer dietary behavior group’, while the remaining three groups were combined to form the ‘better dietary behavior group’. For the exercise-related profiles, the Exercise Dual-Low Group and the Exercise-SS Medium–Low Group were grouped together as the ‘poorer exercise behavior group’, and the other three groups were combined as the ‘better exercise behavior group’. These dichotomized groups were used as the dependent variables in the analyses. The independent variables included in the final logistic regression models are presented.

**Table 5:** Binary logistic regression on insufficient daily exercise.

Variables	*p*	OR	95% CI
Profiles of Exercise-SE and Exercise-SS			
Exercise Dual-Low Group	<0.001	0.046	(0.010, 0.213)
Exercise-SS Medium–Low Group	0.002	0.136	(0.037, 0.495)
Exercise Dual-Medium–Low Group	0.097	0.347	(0.099, 1.213)
Exercise Dual-Medium–High Group	0.642	1.388	(0.349, 5.527)
(Ref. Exercise-SE High Group)			
Age	0.025	1.060	(1.007, 1.115)
Residence			
Urban	0.484	0.787	(0.403, 1.539)
(Ref. Rural)			
Ethnicity			
Han Chinese	0.155	0.523	(0.214, 1.279)
(Ref. Minority)			
Marital status			
Married	0.920	1.114	(0.136, 9.131)
(Ref. Single)			
Education			
9 years or less	0.150	0.566	(0.261, 1.229)
(Ref. 10 years or more)			
Occupation			
Part-time job or no job	0.123	1.632	(0.876, 3.038)
(Ref. Full-time job)			
Monthly income			
≤233 dollars	0.112	1.770	(0.875, 3.581)
(Ref. > 233 dollars)			
Number of pregnancies	0.166	0.842	(0.660, 1.074)
Age of the youngest child			
1–5 years	0.229	1.444	(0.794, 2.629)
(Ref. 6–12 years)			
WC ^a^	0.915	1.002	(0.966, 1.040)
BMI ^b^			
<24.0	0.568	1.281	(0.548, 2.993)
24.0–27.9	0.664	1.193	(0.538, 2.647)
(Ref. ≥ 28.0)			
With any chronic disease			
No	0.057	1.689	(0.984, 2.899)
(Ref. Yes)			
Family history of diabetes			
No	0.458	0.773	(0.392, 1.524)
(Ref. Yes)			
Family history of CVD ^c^			
No	0.798	0.908	(0.434, 1.900)
(Ref. Yes)			
Family history of hypertension			
No	0.711	0.902	(0.522, 1.559)
(Ref. Yes)			

Note: The model adjusted for sociodemographic, anthropometric, and health-related variables. ^a^ WC, waist circumference. ^b^ BMI, body mass index. ^c^ CVD, cardiovascular disease.

## Data Availability

The datasets used during the current study are available from the corresponding author on reasonable request.

## Supplementary Materials

The following supporting information can be downloaded at https://www.mdpi.com/article/10.3390/nu17152478/s1, Supplementary Table S1: Different profiles of diet- or exercise-related self-efficacy and social support; Supplementary Table S2: Exploratory binary logistic regression analysis of insufficient daily fruit/vegetable intake; Supplementary Table S3: Exploratory binary logistic regression analysis of insufficient daily exercise; STROBE checklist: STROBE Statement—Checklist of items that should be included in reports of cross-sectional studies.
