# Supplementary material for: The Profiles of Diet- or Exercise-Related Self-Efficacy and Social Support Associated with Insufficient Fruit/Vegetable Intake and Exercise in Women with Abdominal Obesity
Source: Nutrients. 2025 Jul 29;17(15):2478. doi: 10.3390/nu17152478 (PMC12348066; doi:10.3390/nu17152478)
Supplement: Supplementary file 1 [file nutrients-17-02478-s001.zip › Supplementary Tables.pdf]

Supplementary Table S1

Different profiles of diet- or exercise-related self-efficacy and social support.

Models	Groups	Diet-related self-efficacy	Diet-related social support	Exercise-related self-efficacy	Exercise-related social support
	Overall (n=327)	8.00 [5.00, 10.00]	11.00 [10.00, 12.00]	7.00 [4.00, 9.00]	12.00 [8.00, 14.00]
Diet-related self-efficacy and social support	Diet Dual Low Group (n=18)	1.50 [0, 4.00]	8.00 [6.75, 8.00]		
	Diet SE Medium Group (n=277)	8.00 [5.00, 10.00]	11.00 [10.00, 12.00]		
	Diet SE High Group (n=15)	12.00 [11.00, 16.00]	8.00 [6.00, 8.00]		
	Diet Dual Medium Group (n=17)	10.00 [8.00, 12.00]	16.00 [15.00, 18.00]		
Exercise-related self-efficacy and social support	Exercise Dual Low Group (n=40)			0 [0, 1.00]	6.50 [5.00, 10.00]
	Exercise SS Medium-Low Group (n=92)			4.00 [4.00, 5.00]	10.00 [6.00, 12.00]
	Exercise Dual Medium-Low Group (n=119)			8.00 [7.00, 8.00]	12.00 [10.00, 14.00]
	Exercise Dual Medium-High Group (n=59)			11.00 [10.00, 12.00]	14.00 [12.00, 17.00]
	Exercise SE High Group (n=17)			16.00 [14.50, 16.00]	15.00 [12.00, 19.00]

Supplementary Table S2

Exploratory binary logistic regression analysis of insufficient daily fruit/vegetable intake.

Variables	*P*	OR	95%CI
Profiles of diet-SE and diet-SS			
Diet Dual-Low Group	0.011	0.174	(0.045, 0.673)
(Ref. Diet-SE Medium Group)			
Age	0.241	1.030	(0.980, 1.083)
Residence			
Urban	0.449	1.280	(0.675, 2.427)
(Ref. Rural)			
Ethnicity			
Han Chinese	0.989	0.994	(0.398, 2.480)
(Ref. Minority)			
Marital status			
Married	0.851	0.826	(0.111, 6.120)
(Ref. Single)			
Education			
9 years or less	0.242	0.651	(0.317, 1.337)
(Ref. 10 years or more)			
Occupation			
Part-time job or no job	0.516	1.215	(0.675, 2.186)
(Ref. Full-time job)			
Monthly income			
≤ 233 dollars	0.139	1.668	(0.847, 3.285)
(Ref. ＞ 233 dollars)			
Number of pregnancies	0.119	0.835	(0.666, 1.048)
Age of the youngest child			
1-5 years	0.542	0.839	(0.477, 1.476)
(Ref. 6-12 years)			
WC[a]	0.970	1.001	(0.966, 1.037)
BMI[b]			
<24.0	0.977	0.988	(0.448, 2.180)
24.0-27.9	0.203	0.617	(0.293, 1.298)
(Ref. ≥28.0)			
With any chronic disease			
No	0.013	1.912	(1.149, 3.180)
(Ref. Yes)			
Family history of diabetes			

No	0.294	0.712	(0.378, 1.343)
(Ref. Yes)			
Family history of CVD[c]			
No	0.485	0.780	(0.389, 1.566)
(Ref. Yes)			
Family history of hypertension			
No	0.607	1.147	(0.681, 1.933)
(Ref. Yes)			

Note: Women in the Diet Dual Medium Group (17 participants) and in the Diet SE High Group (15 participants) were excluded in this exploratory analysis. The model adjusted for sociodemographic, anthropometric, and health-related variables.

[a] WC, waist circumference

[b] BMI, body mass index

[c] CVD, cardiovascular disease

Supplementary Table S3

Exploratory binary logistic regression analysis of insufficient daily exercise.

Variables	*P*	OR	95%CI
Profiles of exercise-SE and exercise-SS			
Exercise Dual-Low Group	< 0.001	0.137	(0.047, 0.397)
Exercise-SS Medium–Low Group	0.003	0.397	(0.214, 0.736)
(Ref. Exercise Dual-Medium–Low Group)			
Age	0.114	1.045	(0.989, 1.104)
Residence			
Urban	0.295	0.662	(0.306, 1.432)
(Ref. Rural)			
Ethnicity			
Han Chinese	0.095	0.450	(0.176, 1.150)
(Ref. Minority)			
Marital status			
Married	0.741	1.529	(0.123, 18.959)
(Ref. Single)			
Education			
9 years or less	0.448	0.714	(0.300, 1.703)
(Ref. 10 years or more)			
Occupation			
Part-time job or no job	0.065	1.915	(0.962, 3.813)
(Ref. Full-time job)			
Monthly income			
≤ 233 dollars	0.573	1.261	(0.563, 2.827)
(Ref. ＞ 233 dollars)			
Number of pregnancies	0.072	0.776	(0.589, 1.023)
Age of the youngest child			
1-5 years	0.409	1.318	(0.684, 2.539)
(Ref. 6-12 years)			
WC[a]	0.376	1.018	(0.978, 1.060)
BMI[b]			
<24.0	0.484	1.427	(0.527, 3.869)
24.0-27.9	0.340	1.562	(0.625, 3.904)
(Ref. ≥28.0)			
With any chronic disease			
No	0.213	1.462	(0.804, 2.659)
(Ref. Yes)			
Family history of diabetes			

No	0.384	0.714	(0.334, 1.525)
(Ref. Yes)			
Family history of CVD[c]			
No	0.905	0.952	(0.424, 2.138)
(Ref. Yes)			
Family history of hypertension			
No	0.407	0.773	(0.420, 1.422)
(Ref. Yes)			

Note: Women in the Exercise Dual Medium-High Group (59 participants) and in the Exercise SE High Group (17 participants) were excluded in this exploratory analysis. The model adjusted for sociodemographic, anthropometric, and health-related variables.

[a] WC, waist circumference

[b] BMI, body mass index

[c] CVD, cardiovascular disease
